# Department of Health, Buffalo, N. Y.

**Published:** 1910-10

**Authors:** Francis E. Fronczak

**Affiliations:** Health Commissioner


					﻿Department of Health,
Buffalo, N. Y.
Editor Buffalo Medical Journal-.
Sir—With the increase in the number of cases of anterior
poliomyelitis or infantile paralysis throughout the country, the
department deems it necessary to take precautions to prevent its
spread in this city, if possible.
An immediate report of all cases of this disease or of all
suspicious cases must, therefore, be made to the department from
now on. Although at present the houses will not be placarded,
quarantine must be observed, and attending physicians are re-
quested to give full instructions to the family for the protection
of other children.	Francis E. Fronczak, M. D.,
August 22, 1910.	Health Commissioner.
				

